# The Effect of Acute and Chronic Social Stress on the Hippocampal Transcriptome in Mice

**DOI:** 10.1371/journal.pone.0142195

**Published:** 2015-11-10

**Authors:** Adrian M. Stankiewicz, Joanna Goscik, Alicja Majewska, Artur H. Swiergiel, Grzegorz R. Juszczak

**Affiliations:** 1 Department of Animal Behaviour, Institute of Genetics and Animal Breeding, Polish Academy of Sciences, Jastrzebiec, Poland; 2 Software Department, Faculty of Computer Science, Bialystok University of Technology, Bialystok, Poland; 3 Department of Physiological Sciences, Faculty of Veterinary Medicine, Warsaw University of Life Sciences, Warsaw, Poland; 4 Department of Human and Animal Physiology, Institute of Biology, University of Gdansk, Gdansk, Poland; 5 Department of Pharmacology, Toxicology and Neuroscience, Louisiana State University Health Sciences Center, Shreveport, Louisiana, United States of America; Centro Cardiologico Monzino IRCCS, ITALY

## Abstract

Psychogenic stress contributes to the formation of brain pathology. Using gene expression microarrays, we analyzed the hippocampal transcriptome of mice subjected to acute and chronic social stress of different duration. The longest period of social stress altered the expression of the highest number of genes and most of the stress-induced changes in transcription were reversible after 5 days of rest. Chronic stress affected genes involved in the functioning of the vascular system (*Alas2*, *Hbb-b1*, *Hba-a2*, *Hba-a1*), injury response (*Vwf*, *Mgp*, *Cfh*, *Fbln5*, *Col3a1*, *Ctgf*) and inflammation (*S100a8*, *S100a9*, *Ctla2a*, *Ctla2b*, *Lcn2*, *Lrg1*, *Rsad2*, *Isg20*). The results suggest that stress may affect brain functions through the stress-induced dysfunction of the vascular system. An important issue raised in our work is also the risk of the contamination of brain tissue samples with choroid plexus. Such contamination would result in a consistent up- or down-regulation of genes, such as *Ttr*, *Igf2*, *Igfbp2*, *Prlr*, *Enpp2*, *Sostdc1*, *1500015O10RIK* (*Ecrg4*), *Kl*, *Clic6*, *Kcne2*, *F5*, *Slc4a5*, and *Aqp1*. Our study suggests that some of the previously reported, supposedly specific changes in hippocampal gene expression, may be a result of the inclusion of choroid plexus in the hippocampal samples.

## Introduction

Prolonged stress is an important risk factor for depression [1,2], anxiety [1], drug addiction [3], cardiovascular disease [4,5], ulcers [6], and cancer [7]. Therefore, understanding of the adaptive and maladaptive responses to stressors can unravel the pathogenesis of stress-related diseases. One of the key brain areas involved in stress responses is the hippocampus, which is responsible for the consolidation and retrieval of contextual memories [8]. The relationship between stress and learning is important for several reasons. Stress produces intense and long lasting memories that constitute a source of serious distress [9] and prolonged stress impairs general learning abilities [8].

In order to better understand the effects of stress on the hippocampal formation, we used the microarray technique to detect transcriptomic changes in brains of mice during social stress. Most stressors experienced by humans are of social nature. Therefore, the chronic social stress paradigm in rodents is suggested to be highly relevant for modeling chronic stress in humans. This paradigm allows researchers to study molecular underpinnings of stress in the brain. The microarray technique is a valuable tool for discovering new molecular mechanisms, which are not expected based on current knowledge. An example is the tumor suppressor gene *Fam107a* (*Drr1*), which has been implicated in stress responses by a microarray study and only later has been confirmed in different laboratories applying various stress procedures [10,11,12,13]. However, a serious problem in microarray experiments is the low reproducibility of results, which is especially striking in the case of studies investigating the brain transcriptome [14,15,16]. Previous meta-analysis of pain microarray studies found that only 2% of all reported genes were replicated by 4 independent studies [14]. The difficulty to distinguish the biologically relevant changes from false-positive findings highlights the need for focusing on the replicability of transcriptomic changes detected by microarrays. Therefore, we have investigated gene expression in 4 groups of mice subjected to acute and chronic stress of different duration. Furthermore, we collected not only the hippocampi (present report) but also the frontal cortices [12] to compare the effect of stress on different brain areas collected from the same animals. Such approach may enable the finding of consistent transcriptomic changes that are correlated with the magnitude of the stress and the rejection of poorly replicated results. In order to check the reversibility of the stress-induced transcriptome changes, we also investigated gene expression after a recovery period. Our approach allowed us to identify a number of gene clusters differentially expressed in stress.

## Materials and Methods

### Animals

Outbred, naïve Swiss—Webster male mice (weighing 38.3g ± 0.3g, 12 weeks of age) bred in Institute of Genetics and Animal Breeding, Polish Academy of Sciences were used. They were given ordinary daily care with free access to standard murine dry food (Wytwornia Pasz “Morawski”, Poland) and tap water. Animals were housed in a temperature- (22 ± 1°C) and humidity-controlled (52 ± 2%) room with 12-hour day cycles (lights on at 6 a.m.). Before the start of the experiment the mice were housed three to six per cage. Standard laboratory mouse cages (207 mm × 265 mm and 140 mm high) were made of clear polycarbonate and were covered with stainless steel wire-grid lids that held a feed and water bottle. Softwood granules were used as bedding material. The mice were under constant veterinarian care, and their welfare was assessed during the experiment by measurements of body mass and food consumption. All procedures were performed in accordance with the Guiding Principles for the Care and Use of Research Animals. The study has been approved by the Warsaw Third Local Ethical Committee for Animal Research, which is responsible for the supervision of animal research performed in the Institute of Genetics and Animal Breeding (Permit Number: 37/2009, according to Polish Ministry of Agriculture and Rural Development decree from 10.03.2006 on conditions of maintaining laboratory animals). All efforts were made to minimize the animals’ suffering.

### Experimental procedure

**Fig 1:**
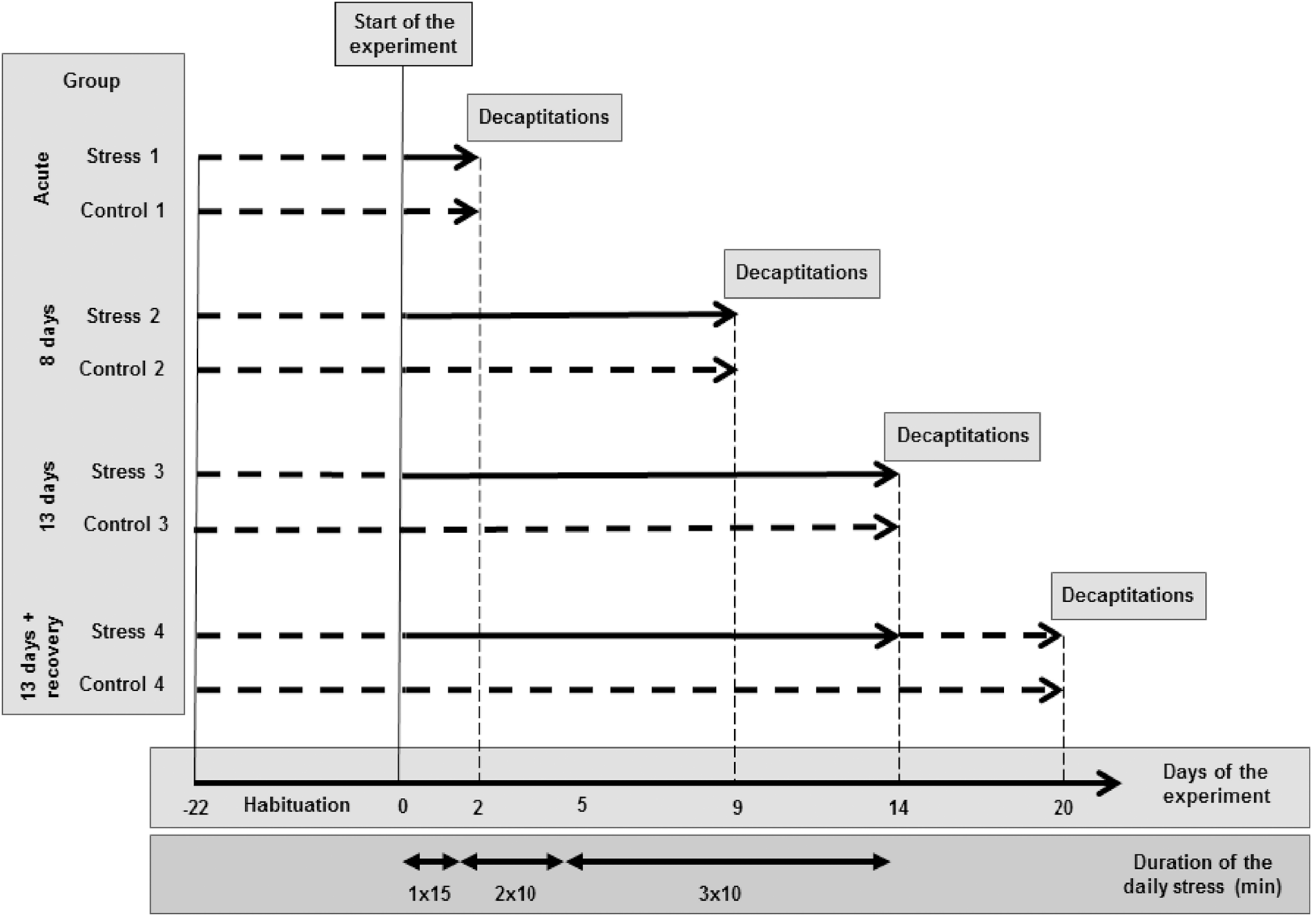
The design of the experiment. Broken lines mark days when a group was not stressed. Solid lines mark days when the group was stressed. The last stress episode was performed 24 h before decapitation.

At the age of 12 weeks the animals were moved from their family cages to individual cages and were single housed until the end of the experiment. Immediately after separation, mice were assigned to one of the stress groups or to the corresponding control groups. Next, they were moved from the main colony room to the behavioral laboratory. The mice designated to stress groups and control groups were kept in separate, adjacent rooms during the entire period of the experiment. The mice were habituated during the first 22 days to their new conditions and next they were subjected to social stress (stress groups) or were left undisturbed (control groups). The duration of habituation was based on observation of food intake [12]. After separation from littermates, the mice displayed cyclic fluctuations in food intake that were associated with the cycle of work of the personnel responsible for the maintenance of the mouse colony. These fluctuations suggest that the separated mice became hypersensitive to environmental stimuli and that they required 3 weeks to fully habituate to new conditions [12]. The mice designated to the stress group were divided randomly into 4 subgroups (n = 12) described in Fig 1. For each stress group, there was a separate control group (n = 12) composed of siblings to enable the comparison between stressed and unstressed brothers. Each stress group and corresponding control group contained at least 10 pairs of siblings derived from different parents.

### Social stress procedure

Social stress was induced by placing a mouse in a cage housing four to five males (4 months old) for 10–15 minutes one to three times per day (10 a.m.– 12 a.m.) (Fig 1). Cages with the group-housed mice were rotated after each social encounter to avoid habituation. We used entire litters as residents to decrease the likelihood of fights leading to injuries and subsequent immune response. It is well known that aggression is increased in single-housed mice and therefore injurious fight is likely between such animals [17]. In contrast, aggression in the group-housed mice is high enough to set the hierarchy but usually does not lead to injuries [18,19]. The duration of a single stress procedure episode (10–15 min) was optimized based on the observation of mice during social encounters. First, the observation was performed to ensure that the procedure induced agonistic behaviors such as upright postures, aggressive grooming, fights, and escape that are characteristic for stressful social encounters in mice [20]. Second, the mice were observed in order to avoid excessive aggression leading to injuries and peripheral inflammation. To protect animals, the duration of social encounters was shortened from 15 to 10 minutes (Fig 1) because there was an increase in aggression on the second day of stress procedure. The frequency of social encounters and the total duration of the stress procedure was based on changes in food consumption during consecutive days of experiment [12]. The applied procedure was sufficient to avoid injures in mice and convenient enough to enable simultaneous processing of all animals belonging to all stressed groups. Twenty-four hours after the last social encounter the mice from the stress groups and the corresponding control groups were decapitated to dissect internal organs (thymus and spleen), which were used as indices of stress response. The effectiveness of the applied stress procedure for the induction of the stress response was confirmed previously [12].

### Sample preparation

After decapitation (11 a.m.– 3 p.m.), brains were removed in order to dissect areas of interest on an ice-cold glass dish. Animals from stressed and control groups were decapitated in an alternating order. To access the hippocampus we first removed the cortex with spatulas, starting from the midline. The entire hippocampi were detached from the fornix with a surgical razor, separated from each other and finally removed. The dissected hippocampi were inserted into freezing vials, frozen in liquid nitrogen and stored at -80°C until further processing. Total RNA was extracted separately from the individual brain samples using NucleoSpin RNA II kit (Macherey-Nagel, Germany) according to the manufacturer's protocol. The quantity and quality of RNA samples were estimated using spectrophotometry (ND-1000, Nanodrop, USA) and microcapillary electrophoresis (Bioanalyzer 2100, Agilent, USA). All of the samples chosen for further analysis were of high quality (RIN >9, 260/280 ~ 2.1).

### Microarray procedures

Nine high quality samples containing the highest amount of isolated RNA were selected from each experimental group. Three independent RNA pools per each experimental subgroup were prepared (24 in total). Each pool consisted of equal amounts of total RNA from 3 mice. 1000 ng of total RNA from each pool was converted to cDNA, then amplified and labeled with cyanine 3 or 5 (Two-Color Quick Amp Labeling Kits, Agilent, USA). The resulting cRNA was hybridized (Gene Expression Hybridization Kit, Agilent, USA) on Agilent's Mouse GE 4x44K microarrays. Siblings from stress and control groups were compared on a single microarray. Six slides containing 24 microarrays were used in the experiment. Twelve microarrays were dye-swaps (analyzing the same samples as those in the ‘original’ microarray, but labeled inversely); these were included to control the unequal fluorescence of the dyes. The data were extracted using G2565CA Microarray Scanner (Agilent, USA) and Agilent Feature Extraction Software (Agilent, USA) on default settings (GE2_1010_Sep10). The study data have been deposited in NCBIs Gene Expression Omnibus (http://www.ncbi.nlm.nih.gov/geo/, accession number GSE59070).

### Real—Time Quantitative PCR

The microarray data were validated utilizing SYBR Green-based real-time quantitative PCR (qPCR) performed in 96-well plates on LightCycler^®^ 480 II thermocycler (Roche, Germany). The hippocampal total RNA samples from the animals used in microarray study were individually analyzed (n = 9; 1 animal from control group for 8 days of stress was missing from analysis due to loss of sample). The expression levels of the following genes were studied: *Ttr*, *Aqp1*, *Prlr*, *Kcne2*, *Folr1*, *Spp1*, *Egr2*, *S100a8*, *Vwf*, and *Gbp4*. We selected genes with high fold change or consistent pattern of expression across the pools of RNA used for microarray analysis. Preference was also given to genes significantly regulated by at least two different stress regimes. On the other hand, *Egr2* was selected because it was one of the few genes specific for the recovery group. *Gapdh* was used as a reference gene because it was the most stably expressed gene from the panel of reference genes (*Gapdh*, *B2m*, *Hprt*, *Sdha*, *Tbp*, *Ywhaz*, *Pgk*) analyzed with NormFinder tool [21]. Primers (see S1 Table for details) were designed using the Primer-BLAST tool (NCBI, Bethesda, USA) with murine RefSeq database. The primers produced amplicons that spanned two exons and included all known alternatively spliced mRNA variants. 500 ng of total RNA from each sample was retrotranscribed to cDNA (First Strand cDNA Synthesis Kit, Roche, Germany). qPCR was run on KAPA SYBR^®^ FAST qPCR Kits (Kapa Biosystems, USA) according to manufacturer’s recommendations. All of the genes were run in triplicate (3 independent runs) except for *Egr2* and *S100a8*, which were run in duplicates. All runs contained a negative control (without cDNA), as well as 5 fold dilution series of cDNA to determine PCR efficiency. A melting curve analysis was performed to verify the presence of one gene-specific peak and the absence of primer-dimer peaks. Raw Ct values were calculated on Lightcycler, using 2’nd derivative method. For each sample the relative expression ratio (R) was calculated according to Pfaffl model [22].

### Data analysis and statistics

#### Real—Time Quantitative PCR and correlation analysis

Variance homogeneity and normality of the data were assessed by Levene's and Lilliefors test respectively. Because PCR data did not meet the requirement of variance homogeneity, the non-parametric Wilcoxon signed-rank test was applied [23,24]. The relationship between gene expression and the respective weight of organs expressed as percentage of body weight was assessed with Spearman's rank correlation coefficient. The effect of stress on the weight of the thymus and spleen (between-group comparison) has been presented in our previous work together with prefrontal microarray data [12]. In the present report the weight of organs is used as an individual measure of stress reactivity. Data analysis was performed with Statistica software, release 7.1. Values are presented as mean ± SEM.

#### Statistical analysis of microarray data

The raw data files were analyzed with the Limma package from the Bioconductor project [25] using the same criteria for all files. Separate channel tests for differential expression, which take under consideration intra-spot correlation, were applied since it has been shown that this approach provides greater statistical power than traditional analysis carried out only in terms of the log-ratios (M-values) [26]. The ‘normexp’ background correction method [27] was used and followed by within-array normalization carried out with the ‘loess’ procedure and between-array normalization conducted with the ‘Aquantile’ method [28]. Both technical (dye-swaps) and biological replicates were taken into consideration while constructing the appropriate design and contrast matrices. The data were analyzed using standard paired analysis that compared each treatment with corresponding control group (samples hybridized to the same arrays). Additionally, a time-course analysis was carried out, also with the use of separate channel approach, which revealed genes showing statistically significant differences in the log fold changes when comparing time periods. To elaborate: for genes with statistically significant positive log fold changes discovered with the time-course analysis (e.g. when comparing time periods time 1 and time 3) the change in expression (stress vs. control) is significantly greater in time period 1. Consequently, the statistically significant negative log fold change discovered with the time-course analysis (e.g. when comparing time periods time 1 and time 3) means, that the change in expression (stress vs. control) is significantly greater in time period 3. Genes showing binary logarithmic fold changes (in their absolute value) greater than 0.5 and adjusted p-values < 0.05 were considered as differentially expressed. P-values were corrected using the Benjamini and Hochberg procedure controlling False Discovery Rate [29]. Using 9 animals per group with 0.05 FDR cutoff allowed us to reach statistical power 0.8 [30].

#### Clustering analysis

Genes that had been found to be significantly up-or down-regulated were included in the clustering analysis. For these genes, binary logarithmic fold changes (logFC) for each technical replicate (original + dye-swapped array) were calculated. LogFC for multiple probes analyzing the same gene were averaged using median. These values were used as an input for clustering. Clustering was carried out with the use of the Cluster 3.0 software (Stanford University, USA) and the results were visualized in Java TreeView [31]. The UPGMA clustering algorithm with absolute centralized correlation as a similarity measure was used [32]. The correlation coefficient (r) > 0.55 was used to assign genes to different clusters. Additionally, we have distinguished subclusters, if the correlation coefficient was equal or higher than 0.75.

### Regional expression analysis using the Allen Brain Atlas

To check if our hippocampal samples were contaminated with adjacent brain tissues, each cluster was screened for specificity of brain distribution using the Allen Brain Atlas (www.alleninstitute.org) [33]. This analysis was prompted by the literature data suggesting that presence of *Ttr* mRNA may be caused by contamination of hippocampal tissue with choroid plexus [34]. Previously, the approach based on the Allen Brain Atlas was used to identify genes constituting likely contamination in microarray profiles of the Purkinje cells [35]. Four representative genes were selected from each cluster to check their spatial distribution following the rules described by [36]. The tested gene was considered as a potential artifact, if it was enriched (displayed high level of expression) in the tissue located next to the hippocampus. After detection of the cluster that could contain contamination, all other genes from the cluster were also tested. Images downloaded from the Allen Brain Atlas were analyzed with ImageJ software (http://imagej.nih.gov/ij/). Regions of interests were determined for each slice based on ISH image while corresponding expression image (expression mask) was used to measure gene expression. The intensity of gene expression in the Allen Brain Atlas is coded by colors that ranges from blue (low expression), through green (medium intensity) to red (high intensity). Mean intensity for each color channel was measured with ImageJ software, multiplied by an integer assigned to each expression level (blue = 1, green = 2, red = 3) [36] and finally summed. The precise anatomical localization of gene expression has been based on the Allen Reference Atlas and literature presenting gene expression in the choroid plexus [37,38].

### Gene annotation

The g:Profiler tool—g:GOSt [39,40] was used for the annotation of gene clusters. The g:Profiler annotates the input gene list using data provided by Gene Ontology (http://www.geneontology.org/), Kyoto Encyclopedia of Genes and Genomes (http://www.genome.jp/kegg/), Reactome (http://www.reactome.org/) and TRANSFAC (http://www.gene-regulation.com/pub/databases.html) databases. Ambiguous gene names were changed to Ensemble (http://www.ensembl.org/index.html) or Mouse Genome Informatics (http://www.informatics.jax.org/) gene IDs for the analysis. G:Profiler analysis was conducted with default settings and was ordered to return only statistically significant results (over-represented functional terms) according to g:SCS multiple testing method [40].

## Results

### General genome-wide expression

**Fig 2:**
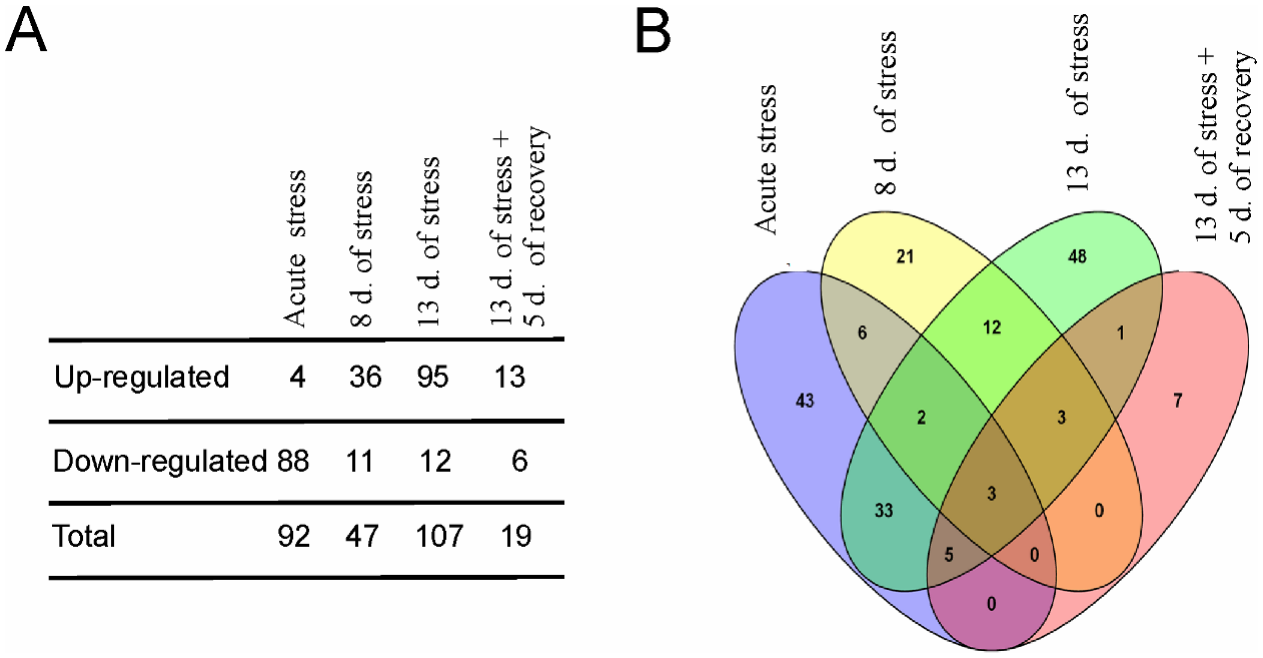
Summary of microarray data. A—total number of significantly regulated genes. B—Venn diagram showing differences and similarities in gene expression between different stress regimes. Each colored ellipse represents one treatment group. Numbers of genes common between treatment groups are depicted on intersections between ellipses.

**Fig 3:**
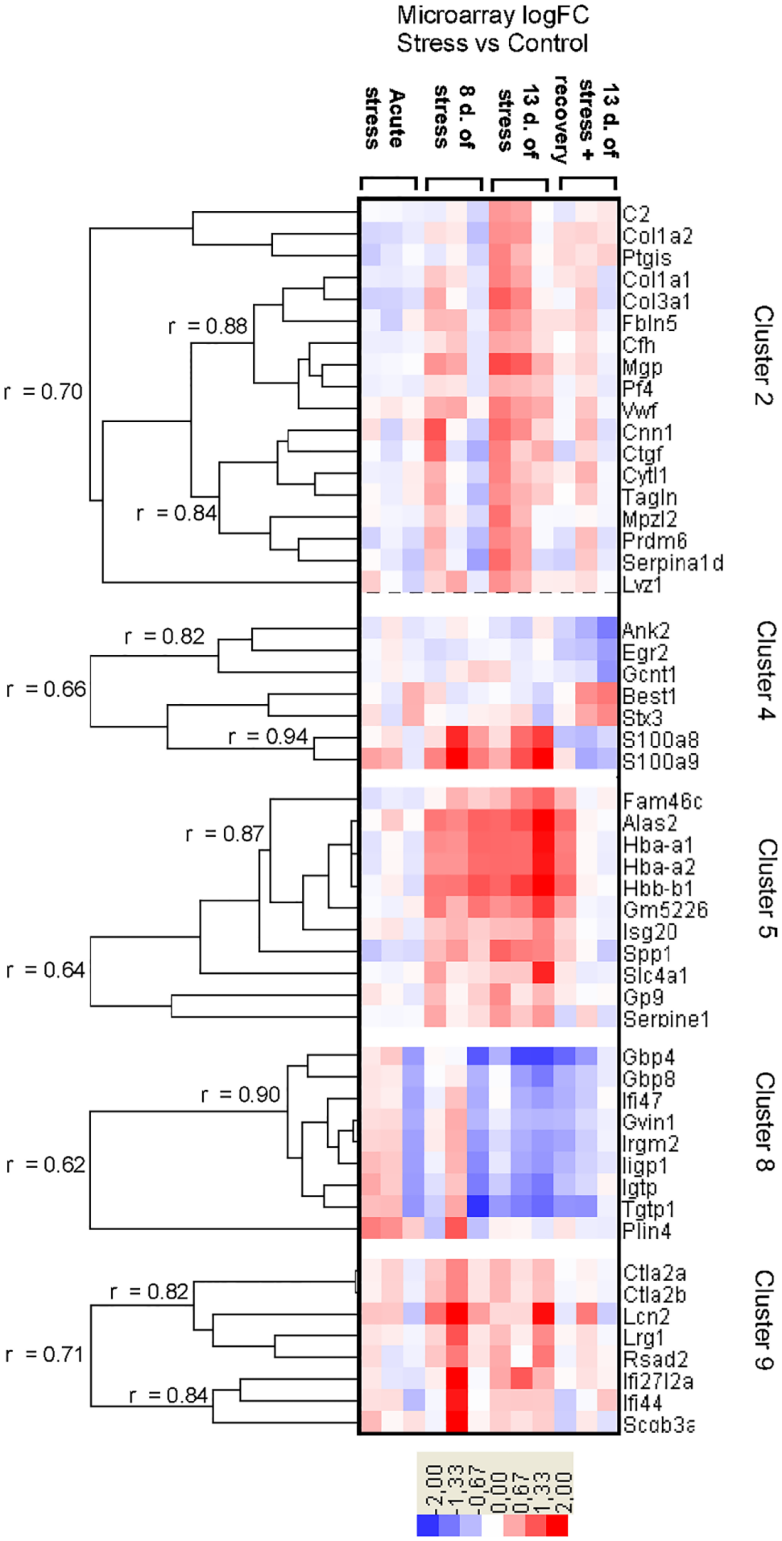
Expression pattern of genes assigned to clusters 2, 4–5 and 8–9. d.- days.

**Fig 4:**
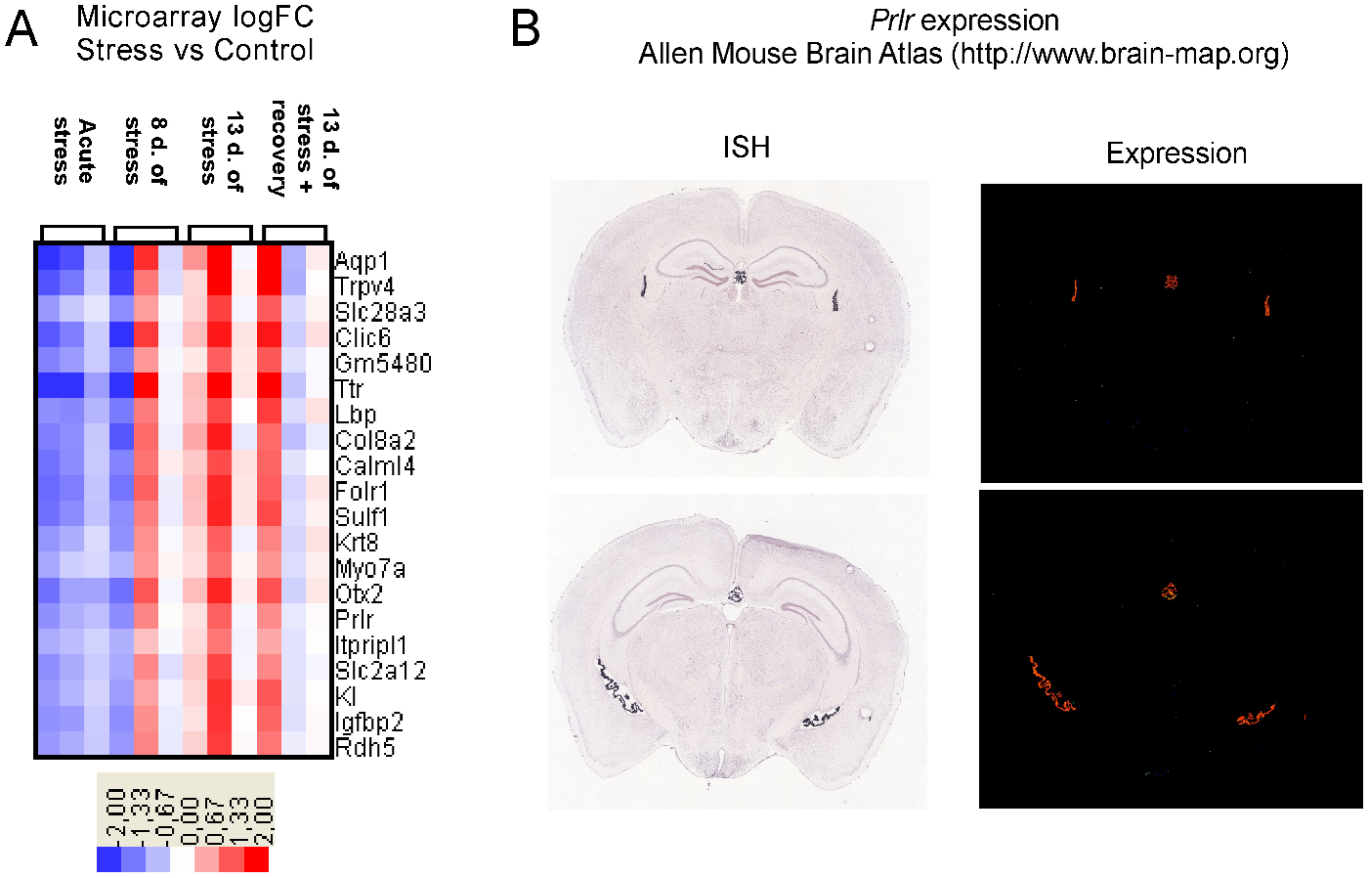
A—expression pattern of genes belonging to cluster 7. B—anatomical pattern of *Prlr* gene expression obtained from the Allen Mouse Brain Atlas. d.- days. Only part of the cluster is presented. For a full list of genes see S2 Table.

**Fig 5:**
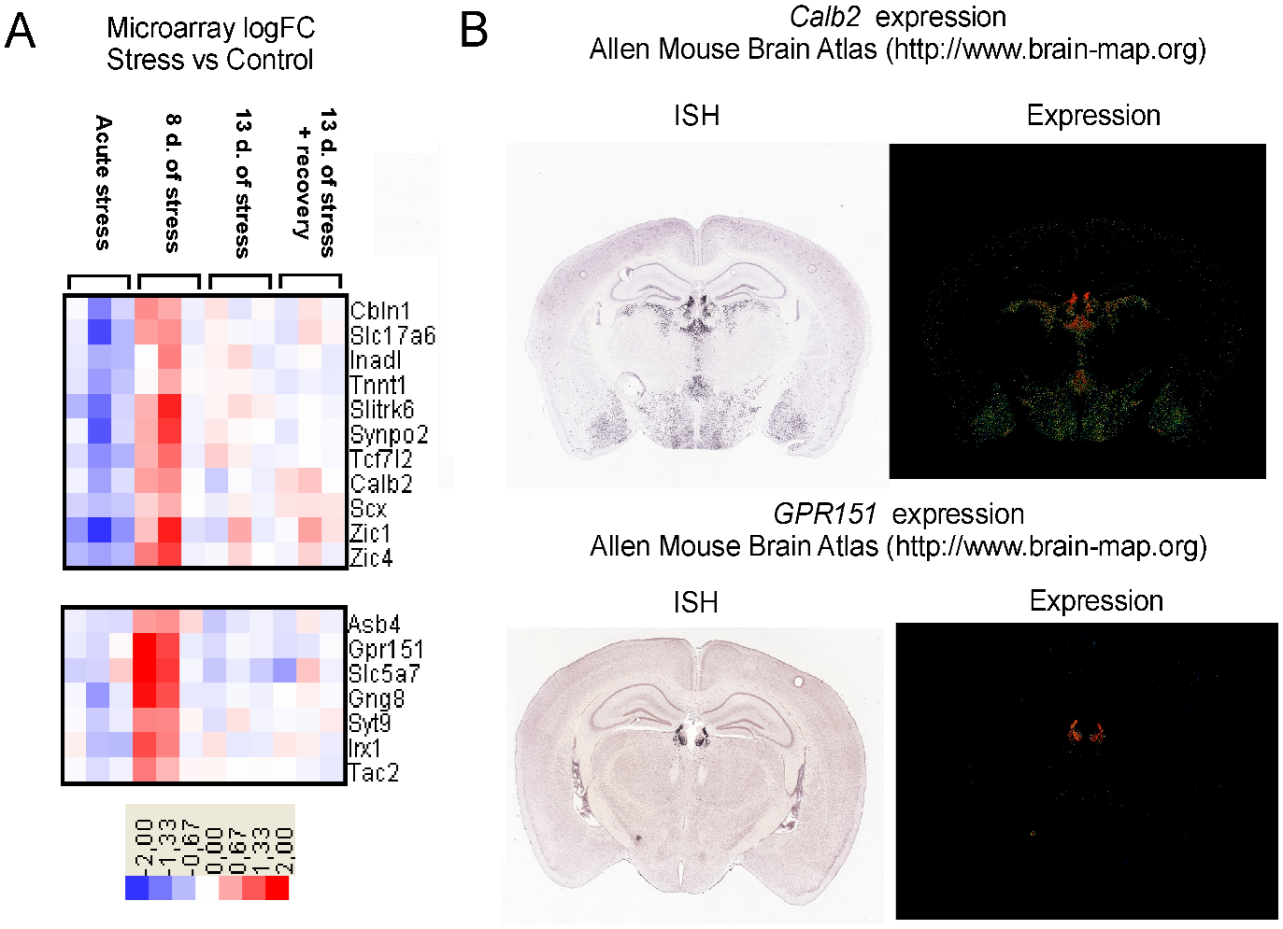
A—expression pattern of genes belonging to clusters 10. B—examples of anatomical patterns of gene expression obtained from the Allen Mouse Brain Atlas. d.- days.

Data were analyzed both using standard paired analysis that compared each treatment with corresponding control group (samples hybridized to the same arrays) and using the time course analysis that compared different treatments (for example acute stress vs. 13 days of stress). A comparison between stress groups and corresponding control groups (paired analysis) revealed 184 genes that were differentially expressed in at least one group of stressed animals. The summary of obtained results is presented in Fig 2 and a full list of significantly regulated probes (as compared to genes showed in Fig 2) can be found in S2 Table. Importantly, the longest period of social stress altered expression of the highest number of genes and most of the stress-induced changes in transcription were reversible after 5 days of rest (Figs 2A and 3). Clustering analysis revealed that significantly regulated genes could be grouped into 11 clusters characterized by distinct patterns of expression (Figs 3–5; S2 Table). The time course analysis revealed 327 genes that were significantly regulated (a list of probes is presented in S3 Table). It confirmed 165 genes (90%) that were detected with paired analysis and revealed 143 new genes. All genes unique for paired analysis (*9430031J16Rik*, *A730090H04Rik*, *AF152371*, *Alb*, *Ank2*, *Crygs*, *Ctla2a*, *Ctla2b*, *Gckr*, *Gcnt1*, *Gp9*, *Gsg2*, *Gusb*, *Gvin1*, *Ifi47*, *Rsad2*, *Scgb3a1*, *Serpine1*, *Tagln*) and 139 genes unique for the time course analysis (97%) had absolute fold change below 1. Only 10 genes unique for the time course analysis were significantly regulated both in animals subjected to 8 and 13 days of stress compared with the acute stress (*Avp*, *Bst2*, *Dynlrb2*, *Fabp7*, *Lgals3bp*, *Mlf1*, *Prkcq*, *Rhod*, *Rnf152*, *Tekt1*).

### Validation of microarray results

11 transcripts were tested using real-time quantitative PCR. Five of these genes (*Ttr*, *Aqp1*, *Prlr*, *Kcne2*, and *Folr1*) belonged to cluster 7, whereas the remaining genes (*Hbb-b1*, *S100a8*, *Spp1*, *Vwf*, *Gbp4*, *Egr2*) belonged to clusters 2, 4, 5, and 8 (Table 1). In case of *Hbb-b1*, *S100a8*, *Spp1*, and *Vwf* there was a relatively low and stable level of expression in control groups and almost all significant differences detected in microarray data were confirmed by PCR analysis (Table 1). The exception was a small increase in the expression of *Hbb-b1* that was detected in the recovery group by one out of four microarray probes (S2 Table). In the case of *Gbp4* there was a significant decrease in the recovery group confirming the microarray data. In two other groups, there were trends in the expression of *Gbp4* that were consistent with the microarray results. The PCR data also confirmed the lower level of *Egr2* in the recovery group (Table 1). Therefore, the validation rate of genes belonging to clusters 2, 4, 5, and 8 was comparable to the general trend reported in the literature [41,42]. An opposite situation was found in the case of genes that belonged to cluster 7 (*Ttr*, *Aqp1*, *Prlr*, *Kcne2*, and *Folr1*) as only an increased level of *Aqp1* in one group (Table 1) was confirmed. All remaining differences were not significant, although the expression displayed trends consistent with the microarray data (S4 Table). It is worth noting that the expression of all these genes was characterized by the lack of a stable baseline in control groups.

**Table 1:** Quantitative qPCR validation of microarray data.

Duration of stress	Recovery period	Gene Name	Cluster	Microarray logFC	Microarray P value	qPCR logFC	qPCR P value
Acute	_	*Aqp1*	7	-1.43	< 0.0001	-1.84	NS
Acute	_	*Ttr*	7	-1.95	< 0.0001	-1.89	NS
8 days	_	*Gbp4*	8a	-0.55	0.0009	-0.62	NS
8 days	_	*Hbb-b1*	5a	1.15	< 0.0001	1.4	0.01
8 days	_	*S100a8*	4b	0.9	0.02	1.91	0.04
8 days	_	*Spp1*	5a	0.55	0.01	0,4	NS
8 days	_	*Ttr*	7	-0.54	0.0002	-0.29	NS
13 days	_	*Aqp1*	7	1.18	< 0.0001	1.81	0.03
13 days	_	*Gbp4*	8a	-1.47	< 0.0001	-0.91	NS
13 days	_	*Hbb-b1*	5a	1.69	< 0.0001	1.95	0.008
13 days	_	*S100a8*	4b	0.98	0.004	1.94	0.04
13 days	_	*Spp1*	5a	1.06	< 0.0001	1.29	0.008
13 days	_	*Ttr*	7	1.22	< 0.0001	1.69	NS
13 days	_	*Vwf*	2b	0.81	< 0.0001	0.76	0.02
13 days	5 days	*Aqp1*	7	0.66	0.0001	0.06	NS
13 days	5 days	*Egr2*	4a	-0.68	< 0.0001	-0.63	0.008
13 days	5 days	*Gbp4*	8a	-0.89	< 0.0001	-0.99	0.02
13 days	5 days	*Hbb-b1*	5a	0.45	0.0002	0.23	NS
13 days	5 days	*Ttr*	7	0.78	< 0.0001	0.16	NS

Wilcoxon test, compared with corresponding control group.

### Regional expression analysis using the Allen Brain Atlas

**Fig 6:**
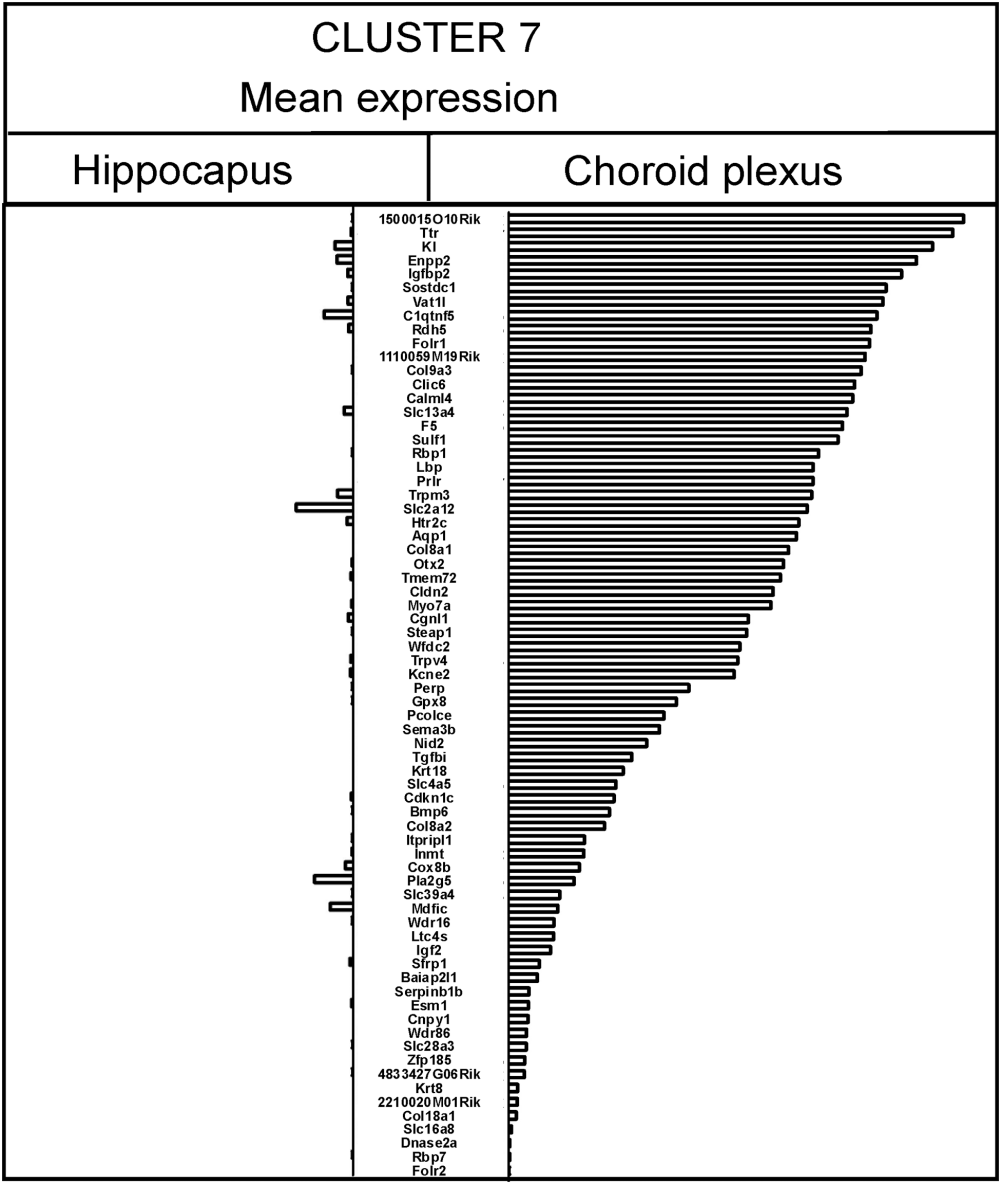
Expression of genes belonging to cluster 7 in hippocampus and choroid plexus based on the Allen Brain Atlas. The intensity of gene expression in the Allen Brain Atlas is coded originally by colors. The average level of each transcript is expressed as a sum of mean intensities for blue, green and red channel multiplied by an integer representing different levels of expression in the Allen Brain Atlas.

The analysis revealed that two clusters contain genes, whose expression is enriched in tissues located in the vicinity of the hippocampus according to the Allen Brain Atlas. Cluster 7 is characterized by the presence of genes enriched in the choroid plexus (Figs 4B and 6). Out of 76 genes from this cluster, 20 are not expressed in the hippocampus while the remaining genes display various hippocampal expression located mainly in the pyramidal or granule cell layers. In all cases the average expression within the entire hippocampal area is lower than in the choroid plexus (Fig 6). Five genes from this cluster (*2900017F05Rik*, *AK048085*, *Gm5480*, *Tuba1c*, *Vpreb1*) are not present in the Allen Brain Atlas.

The cluster 10 (Fig 5A) contain genes, whose expression is enriched in the midline structures such as habenula and septal nuclei. The subcluster 10a contains 5 genes (*Eomes*, *Ebf3*, *Sln*, *Postn*, *Gpr88*) that are expressed in septal nuclei (triangular or lateral nucleus), the subcluster 10b contains genes that are expressed in habenula (*Gpr151*, *Slc5a7*, *Syt9*, *Irx1*, *Tac2*) while the subcluster 10c contains genes that are expressed both in septal nuclei and habenula (*Cbln1*, *Slc17a6*, *Calb2*, *Zic1*) or only in habenula (*Tcfl2*) (S1 Fig). Other genes (*Ebf2*, *Scn5a*, *Gusb*, *Inadl*, *Scx*, *Tnnt1*, *Slitrk6*, *Synpo2*, *and Zic4*) displayed much lower mean expression in all tested areas (S1 Fig) or were not available in the Allen Brain Atlas (*Ctxn3*, *9430085L16Rik*, *1500016L03Rik*, *and Gng8*).

### Annotation enrichment of clusters

Clusters were annotated with information on biological processes, molecular functions, cellular components, biochemical pathways enriched in cluster and hypothetical regulatory factors (S5 Table), as well as basal gene expression levels in brain areas, immune cells and other selected tissues (S6 Table). Briefly, genes belonging to cluster 9 are highly expressed in microglia and immune cells, as compared to neuronal tissue. Genes comprising cluster 8 participate in the response to cytokines, and more specifically, to interferon-gamma and beta, and are regulated by IRF transcription factor. The expression of these genes is higher in immune cells compared to brain. Genes comprising cluster 4 and 4b are involved in processes, such as long-chain fatty acid binding, neutrophil aggregation and chemotaxis. Genes comprising cluster 5 participate in forming hemoglobin complex, O2/CO2 exchange in erythrocytes, immune system process and anemias. Genes comprising cluster 2 are involved in cell adhesion molecule binding, connective tissue development, complement and coagulation cascades, extracellular matrix organization, blood vessel development, extracellular matrix-receptor interaction, integrin binding and response to wounding. Genes comprising cluster 2a may be regulated by miR-877 and are connected with response to wounding, connective tissue development, integrin binding, platelet activation and aortic dilatation.

### Correlation between gene expression (PCR) and organ weights

The characteristic pattern of stress-induced changes consists of the decreased weight of the thymus and the increased weight of the spleen [12,43,44]. The analysis revealed that there was significant negative correlation between the thymic weight and the expression of *Hbb-b1*, *S100a8* and *Vwf* while the spleen weight was positively correlated with theexpression of *Hbb-b1*, *S100a8*, *Vwf*, and *Gbp4* (Table 2). The positive correlation between the spleen weight and *Gbp4* was unexpected because the expression of this gene was lower in chronically stressed animals. However, an analysis performed separately for each stress regime revealed that there was significant correlation only in animals subjected to acute stress (r = 0.68, p = 0,002). Genes assigned to the choroid plexus cluster were not correlated with the weight of the thymus and the spleen (Table 2). More detailed information about changes in the weight of organs in the animals used in this experiment are available in our previous work describing transcriptomic changes in the prefrontal cortex [12].

**Table 2:** Spearman's rank correlation between individual gene expression (PCR) and indices of stress.

Gene Name	Thymus	Spleen
*Hbb-b1*	**r = -0.44; p < 0.001**	**r = 0.27; p < 0.05**
*Spp1*	r = -0.22; p = 0.06	r = 0.19; p = 0.10
*S100a8*	**r = -0.32; p < 0.01**	**r = 0.35; p < 0.01**
*Vwf*	**r = -0.38; p < 0.01**	**r = 0.24; p < 0.05**
*Gbp4*	r = 0.23; p = 0.06	**r = 0.43; p < 0.001**
*Egr2*	r = -0.01; p = 0.92	r = -0.03; p = 0.83
*Ttr*	r = 0.02; p = 0.88	r = -0.07; p = 0.57
*Aqp1*	r = -0.09; p = 0.44	r = 0.01; p = 0.95
*Prlr*	r = -0.10; p = 0.39	r = -0.04; p = 0.74
*Kcne2*	r = -0.07; p = 0.57	r = -0.08; p = 0.52
*Folr1*	r = 0.02; p = 0.85	r = -0.04; p = 0.76

Correlation has been calculated for pooled data from all acute and chronic stress groups.

## Discussion

### Biological function of stress related genes

The most striking effect of social stress was the increase in the expression of genes coding for hemoglobin (*Hbb-b1*, *Hba-a1*, *Hba-a2*) together with up-regulation of *Alas2* (hem biosynthesis) and hemoglobin pseudogene *Gm5226*. All these genes were up-regulated both in animals subjected to 8 and 13 days of stress, their fold changes were higher than 1 (S2 and S3 Tables) and the differential expression of *Hbb-b1* was confirmed by PCR analysis (Table 1). The expression of *Hbb-b1* was also correlated with the indices of stress reactivity (Table 2). After 5 days of recovery, the expression of these genes returned to the baseline. Previously, stress-induced up-regulation of hemoglobin genes *(Hbb-b1*, *Hba-a1*) was also reported in nucleus accumbens, ventral tegmental area, prefrontal cortex and the hippocampus of stressed mice [12,45,46] and both in the prefrontal cortex and the hippocampus of chronically restrained pigs [47]. The only chronic stress study that reported down-regulation of hemoglobin genes in rats was published by Andrus et al. [48]. It is known that hemoglobin is expressed in neurons [49], plays an important role in neuronal respiration [50] and is up-regulated in different pathological states such as intracerebral hemorrhage [51] and ischemia [52]. Therefore, stress-induced up-regulation of these genes may result from changes in the function of vascular system.

**Fig 7:**
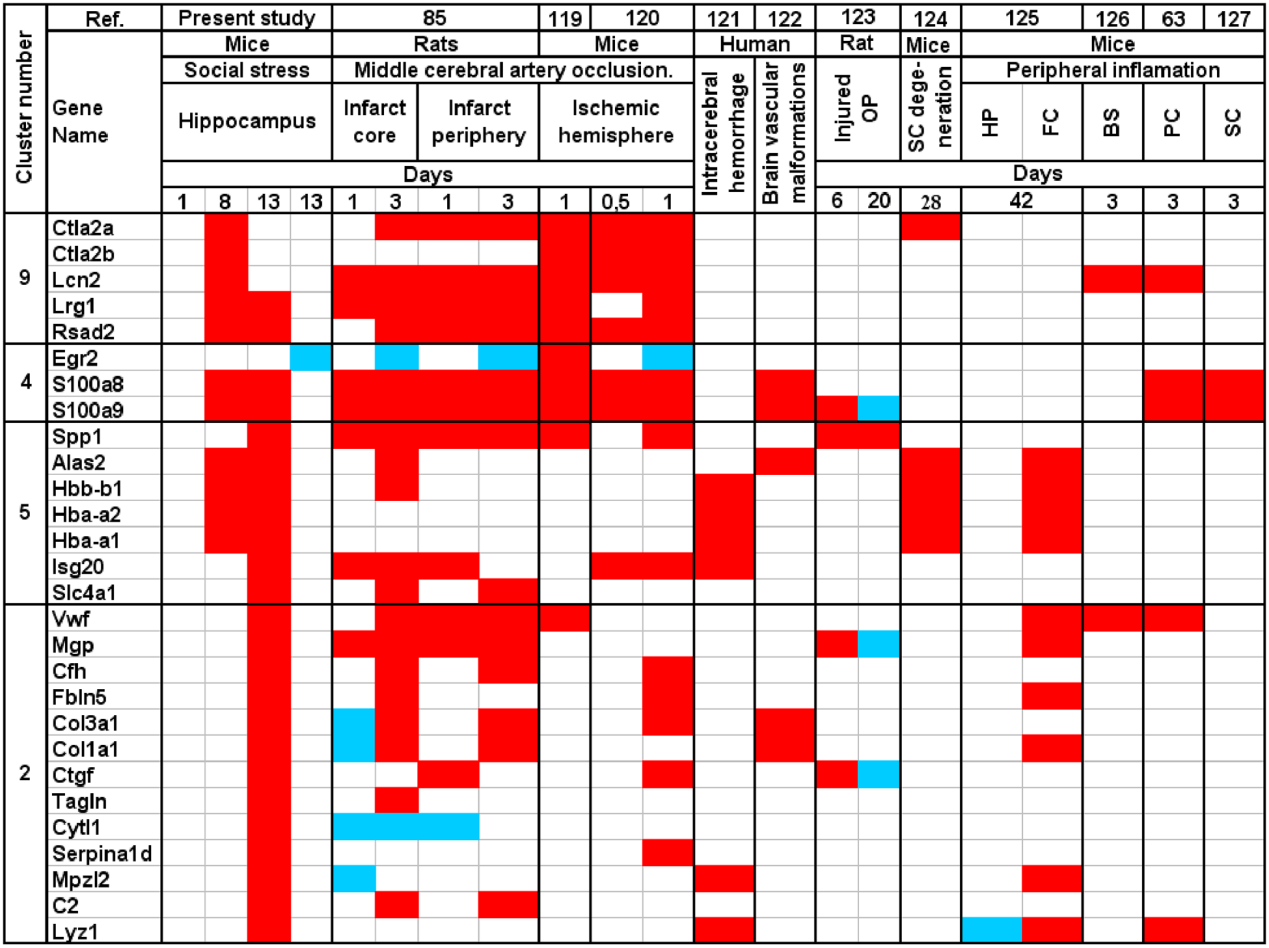
Comparison of gene expression induced by social stress and various kinds of injury. Red—up-regulation, blue—down-regulation, op—olfactory pathway, sc—spinal cord, hp—hippocampus, fc—frontal cortex, bs—brain stem, pc—prefrontal cortex. The data are based on present experiment and previous transcriptomic studies [63,85,119–127].

Other genes belonging to this cluster (5a) were significantly regulated after 13 days of stress, when hemoglobin genes achieved the highest level of expression (S2 and S3 Tables). *Spp1*, had fold change higher than 1 (S2 and S3 Tables) and the differential expression of this gene was confirmed by a PCR analysis (Table 1). *Spp1* plays an important role in processes underlying tissue repair such as collagen remodeling, promotion of the migration of monocytes/macrophages and regulation of inflammatory activity during wound healing [53,54]. *Spp1* expression is increased following brain infarct [55] and in vessels with blood—brain barrier impairment [56]. Injury-induced pattern of co-expression of *Spp1* and other genes is shown in Fig 7. In the microarray studies of stress response, *Spp1* was consistently up-regulated after social stress (present study), recall of contextual fear conditioning [57] and 7 day-long immobilization [58]. The increased expression was also observed in highly-anxious rats [59]. *Fam46c* (Fold change > 1) *and Isg20* are interferon-responsive genes involved in inflammatory processes [60,61]. The up-regulation of *Isg20* has been found after different kinds of brain injury (Fig 7). Summing up, genes belonging to cluster 5a are involved in the functioning of vascular system, vascular injury and inflammation. The up-regulation of these genes suggests that chronic social stress had adverse effect on brain vascular system.

Hemoglobin genes (cluster 5a) were also closely correlated with the expression of genes belonging to cluster 4, which contained two functionally related genes (*S100a8* and *S100a9*) that were up-regulated both by 8 and 13 days of stress. The fold change was close to (*S100a8*) or higher (*S100a9*) than 1 and the differential expression of *S100a8* was confirmed by PCR analysis (Table 1). Furthermore, the expression of *S100a8* was significantly correlated with the weight of the thymus and the spleen (Table 2), which play an important role in the immune system and are affected by chronic stress [12,44]. Stress-induced up-regulation of *S100a8* and *S100a9* was not reported previously in mouse hippocampus although such changes were found in rats subjected to restraint stress and fear conditioning [48] and in ventral tegmental area of stressed mice [45]. S100A8 and S100A9are calcium-binding proteins involved in the regulation of immune cell transmigration during inflammatory response [62] and are up-regulated in different pathological states (Fig 7). The available data show that the up-regulation of *S100a8* and *S100a9* results from the migration of activated neutrophils into the brain [63]. These results are consistent with recent studies showing that repeated social defeat promotes migration of leukocytes to the brain [64]. Other genes from cluster 4 (Fig 3) were significantly regulated during the recovery period following the stress and had low fold change (*Ank2*, *Egr2*, *Gcnt1*, *Best1*, *Stx3*). *Best1* and *Ank2* modulate Ca^2+^ signaling [65,66], *Stx3* is involved in chemokine release and cell transmigration [67], while *Gcnt1* is involved in the trafficking of leukocytes [68]. Summing up, changes in the expression of genes belonging to cluster 4 suggest that chronic social stress is associated with migration of immune cells into the brain vascular system.

In addition to hemoglobin genes and *S100a8 /9*, there were also consistent changes in the expression of genes belonging to cluster 9 (Fig 3). Four genes (*Lcn2*, *Lrg1*, *Rsad2*, *Ifi27l2a*) were consistently up-regulated both in animals subjected to 8 and 13 days of stress, while four additional genes (*Ctla2a*, *Ctla2b*, *Ifi44*, and *Scgb3a1*) were up-regulated only in animals subjected to 8 days of stress (S2 Table). The most prominent was the increase in the expression of *Lcn2*, which had a fold change higher than 1 (S2 and S3 Tables). Previously, stress-induced changes in the expression of these genes were not reported in mouse hippocampus although similar changes in the expression of *Lcn2* and *Lrg1* were found in rats after the recollection of contextual fear memories [57] and in nucleus accumbens and ventral tegmental area of stressed mice [45]. *Lcn2* is known to regulate the migration and adhesion of neutrophils [69], while *Lrg1* mediates neutrophil differentiation [70]. The expression of *Ctla2a*, *Ctla2b* and *Scgb3a1 (Ugrp2)* is up-regulated by interleukin-4 [71,72]. Previous microarray experiments showed that the brain expression of these genes is increased by impaired blood flow (Fig 7). Summing up, cluster 9 contains up-regulated genes that are expressed at high level in microglia, macrophages and other immune cells (S6 Table) and are involved in inflammatory response. Up-regulation of these genes is consistent with up-regulation of *S100a8* and *S100a9* belonging to cluster 4.

Cluster 8 (Fig 3) contains highly correlated genes constituting interferon gamma (IFNγ) responsive GTPases such as *Gbp4*, *Gbp8*, *Ifi47* (*Irg-47*), *Gvin1* (*Vlig-1*), *Irgm2*, *Iigp1*, *Igtp*, and *Tgtp1* (*Tgtp*) [73,74,75]. In our experiment, chronic stress decreased the expression of these genes suggesting a down-regulation of interferon gamma signaling. PCR analysis showed that *Gbp4* was down-regulated in two groups of animals subjected to 13 days of stress although the level of significance was achieved only in the group sacrificed after 5 days of the recovery period following chronic stress. The release of IFNγ plays a crucial role in defense against infections but it also inhibits the synthesis of collagen type I and III [76]. Therefore, the decrease in interferon gamma signaling is consistent with increased expression of collagen that was also found in our study. Production of interferon gamma is decreased by physical injury [77] and psychological stress [78]. The mechanisms responsible for the curbing of the synthesis of interferon gamma include release of stress hormones that additionally stimulate synthesis of interleukin-4 (IL-4) and other cytokines produced by Th2 lymphocytes [78,79]. Therefore, up-regulation of genes belonging to cluster 9 (IL-4 responsive) and down-regulation of cluster 8 (IFNγ responsive) is consistent with the known effects of stress on the immune system.

**Fig 8:**
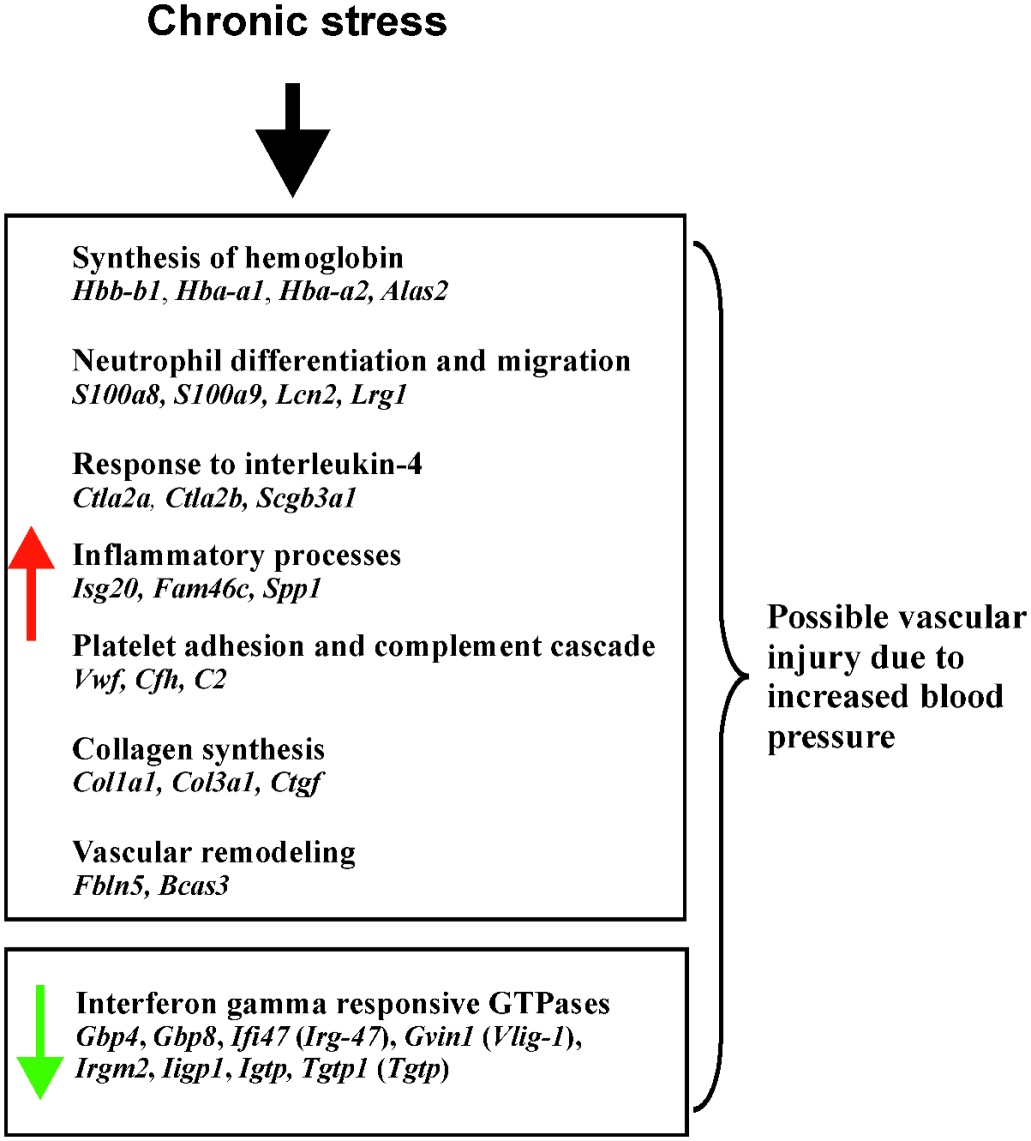
Summary of transcriptomic changes induced by chronic stress.

Cluster 2 (Fig 3) contains 18 genes that were up-regulated only in one group of mice subjected to 13 days of chronic stress. PCR analysis confirmed the increased expression of the Von Willebrand factor (*Vwf*), which was correlated with indices of stress (Table 2). The Von Willebrand factor plays an important role in platelet adhesion to wound sites [80]. Connective tissue growth factor (*Ctgf*) stimulates collagen synthesis [81]and together with *Col1a1* and *Col3a1*, plays a crucial role in wound healing [82,83]. In microarray studies of stress, expression of *Col1a1* was both increased (present study, [58]) and decreased [48,57,84]. This pattern is consistent with the biphasic effect of injury on collagen expression in the brain [85] (Fig 7). The complement component *C2* participates in the activation of the complement cascade and regulates vascular permeability [86] while complement factor H (*Cfh*) protects tissues from damages [87]. Both *C2* and *Cfh* are up-regulated after stroke [85] together with other genes from this and other clusters (Fig 7). Hypoxia activated protein Fibulin 5 (*Fbln5*) is essential for elastic fiber assembly during vascular remodeling and controls endothelial cell adhesion, motility, and proliferation [88,89], while *Bcas3* controls directional cell migration during angiogenesis [90]. Finally, *Mgp* (matrix Gla protein/*Mglap*) plays a homeostatic role in preventing pathological calcification of vessels in response to an increased level of calcium [91] while calponin (*Cnn1*) is involved in the regulation of vascular smooth muscle tone [92]. Summing up, up-regulated genes belonging to this cluster are functionally connected with the vascular system and injury response. Processes associated with these genes include collagen synthesis, platelet adhesion and activation of the complement cascade (Fig 8).

### Genes detected in time course analysis

Apart from the genes that showed significant differences in expression between stressed and control groups in specific time points, our time course analysis has identified genes, whose relative expression was significantly different when comparing two time points. The performed analysis confirmed 165 genes (90%) that were detected with paired analysis (comparison between treatment groups and corresponding control groups) and revealed 143 new genes. Almost all genes unique for the time course analysis (97%) had relatively low fold change. This analysis revealed a number of new genes specific for the vascular system and injury response such as hemolytic complement (*Hc*), angiopoietin 2 (*Angpt2*), fibroblast activation protein (*Fap*), myoferlin (*Myof*) and vascular cell adhesion molecule 1 (*Vcam1*). However, only 10 genes unique for the time course analysis were significantly regulated both in animals subjected to 8 and 13 days of stress compared with the acute stress (*Avp*, *Bst2*, *Dynlrb2*, *Fabp7*, *Lgals3bp*, *Mlf1*, *Prkcq*, *Rhod*, *Rnf152*, *Tekt1*). Below, we discuss two most interesting genes from this group. For a full list of genes differentially expressed in the time course analysis, see S3 Table.

Arginine vasopressin (*Avp*) has been found to affect anxiety [93], memory [94] and may protect brain cells from excitotoxicity [95]. AVP innervation to hippocampus originates mainly from the paraventricular nucleus of hypothalamus (PVN) [96] controlling the release of stress hormones. A possible source of increased level of *Avp* transcripts in the hippocampal tissue may be the axonal transport of mRNA from the PVN because *Avp* mRNA is up-regulated by stress in PVN [97] and is transported into axons [98]. *Fabp7* gene encodes the brain fatty acid binding protein that regulates hippocampal functions such as neurogenesis and behavior [99] and may be associated with psychiatric disorders [100,101]. Stress-induced changes in expression of *Fabp7* were not reported previously but there are data showing increased expression in injured brain [102].

### Markers of tissue cross contamination

An effort has been made to identify and quantify contamination using genomic biomarkers in medical diagnostics [103,104] but this problem is poorly recognized in genomic studies of the nervous system. Previously, it has been suggested that *Ttr* expression in brain tissues results from contamination of samples with choroid plexus [34] but this finding has not been employed in assessment of microarray data. One of the reasons may be the fact that a single gene may not be suitable to judge the quality of samples that are analyzed with a technique yielding a considerable number of false positive findings. Our cluster analysis performed on a large number of independent microarray comparisons revealed that the hippocampal expression of *Ttr* is highly correlated with a number of other genes that are enriched in choroid plexus according to the Allen Brain Atlas (Fig 4A). Some of these genes (*Igf2*, *Igfbp2*, *Clic6*, *Sostdc1*, *Enpp2*, *Kl*, *1500015O10RIK*, *Ttr*, and *Cgnl1*) belong to the group of the most highly expressed transcripts constituting the top 0.5% of transcribed genes in mouse choroid plexus [105]. The choroid plexus specificity was independently confirmed in case of *Ttr* [34,37] and *1500015O10RIK* (*Ecrg4*) [38], whereas marked enrichment was found in the case of *Kl* [37], *Clic6* [37], *Kcne2* [106], and *Htr2c* [107]. Importantly, choroid plexus genes (*Ttr*, *Aqp1*, *Prlr*, *Kcne2*, *Folr1*) were not correlated with indices of stress in contrast to other genes such as *Hbb-b1*, *S100a8*, *Vwf*, and *Gbp4*. The identification of the entire cluster of genes specific for the choroid plexus increases the chance to accurately assess contamination in microarray data. An analysis of already published microarray experiments showed that consistent up-or down-regulation of choroid plexus genes (for example *Ttr*, *Sostdc1*, *Slc4a5*, *Prlr*, *Kl*, *F5*, *Enpp2*, *Clic6*, *1500015O10RIK*, *Otx2*, *Aqp1*) is present also in other studies investigating changes in stress-induced gene expression in the hippocampus [48,108,109] and the amygdala [110]. Further information can be obtained from GEO database (http://www.ncbi.nlm.nih.gov/geo/), which yields 137 gene profiles after entering “*Ttr*” and “hippocampus” as a keywords. An inspection of some of the GEO profiles (for example GDS3598, GDS4413, GDS3479) revealed the same pattern of expression characterized by a high correlation of genes specific for choroid plexus such as *Ttr*, *Sostdc1*, *Slc4a5*, *Prlr*, *Kl*, *F5*, *Enpp2*, *Clic6*, *1500015O10RIK*, *Otx2*, *Aqp1* and other (clustering with euclidean complete linkage). This suggests that the inclusion of the choroid plexus in the hippocampal samples is common. A previous experiment showed that gross dissections of the hippocampus yielded considerable level of *Ttr* mRNA even when attempts have been made to remove the choroid plexus and only microdissection produced samples without *Ttr* expression [34]. The choroid plexus is present in all brain ventricles [111] and, therefore, contamination with this tissue is possible during the dissection of different parts of the brain. Also genes from cluster 10 that are connected with the habenula and septal nuclei have been reported in previous hippocampal studies [13,46,108]. These data suggest that imprecision in tissue dissection is an important factor affecting molecular analyses performed on homogenized samples collected from rodent brains.

### Technical Limitations

The limitation of this study is the design applying sample pooling. Such approach allows to decrease the costs of the experiment but it also decreases the accuracy of the analysis when a small number of subjects contribute to each pool [112]. The decreased accuracy results from the decreased statistical power, the inability to estimate the within population variation and from the increased effect of outliers on data obtained with pooled designs [112,113]. Experimental comparison of the pooled and non-pooled designs with a small number of used animals (3–5 per group) revealed that the analysis based on individual samples allows detection of larger number of differentially expressed genes [114]. On the other hand, pooled and non-pooled designs yielded similar results when clustering methods were used despite the large difference in total number of detected genes [114]. There was also the same time dependence of the transcriptional response within the pooled and individual analyses [114]. Therefore, the experimental design that was used in our study has a potential to indicate true transcriptomic changes although it should be assumed that a considerable number of significantly regulated genes were not detected in our study. It is worth to notice that experimental design with a small number of pooled samples (2–3) was used frequently in the past [41,58,115,116,117] and the limitations associated with such designs can contribute to considerably small overlap between results obtained in different experiments. To decrease the number of false negative and positive errors, it is currently advised to analyze individual samples or to increase the number of pooled subjects [112,114].

## Conclusions

The stress-induced up-regulation of *S100a8*, *S100a9*, *Lcn2*, *Lrg1*, *Rsad2*, and *Ifi27l2a* in mouse hippocampus was reported for the first time. Another prominent group of transcripts were genes involved in synthesis of hemoglobin (*Alas2*, *Hbb-b1*, *Hba-a2*, *Hba-a1)*. The expression pattern of these genes was not unique for the hippocampus because very similar transcriptomic changes have been found in the prefrontal cortex [12]. We also report the increased expression of the Von Willebrand factor (*Vwf*) that was correlated with the indices of stress and decreased expression of interferon gamma (IFNγ) responsive GTPases. The impaired IFNγ signaling can decrease the ability of the immune system to protect organism against infections. The summary of obtained results is presented in Fig 8. These data point to the regulation of genes involved in the function of vascular system, injury and inflammation and suggest that the vascular system may constitute a link between stress and stress-induced brain pathology. A mechanism responsible for vascular dysfunction may include sharp increases in blood pressure leading to endothelial injury, inflammation, compromised blood flow, and impaired blood-brain barrier [12]. This conclusion is consistent with a growing amount of data linking vascular dysfunction with psychiatric diseases such as depression [118].

Furthermore, our data suggest for the first time that changes in expression of some genes that were previously reported to be regulated by stress (for example *Sostdc1*, *Slc4a5*, *Prlr*, *Kl*, *F5*, *Enpp2*, *Clic6*, *1500015O10RIK*, *Otx2*, *Aqp1*) may have resulted from contamination of the brain neural samples with the choroid plexus. These genes can be used as biomarkers of the choroid plexus. In future research it will be important to apply both pre-and post-experimental quality assessments based on biomarker genes and to replace gross dissections with microdissections. It also means that there is a need for further research to ensure precise anatomical localization of stress-induced changes in hippocampal transcriptome.

## Supporting Information

S1 Fig Expression of genes belonging to cluster 10 in hippocampus, habenula, septal nuclei and choroid plexus based on the Allen Brain Atlas. The intensity of gene expression in the Allen Brain Atlas is coded originally by colors. The average level of each transcript is expressed as a sum of mean intensities for blue, green and red channel multiplied by an integer representing different levels of expression in the Allen Brain Atlas.
(TIF)

S1 Table Primers for qPCR. (XLSX)

S2 Table List of probes altered by social stress. (XLSX)

S3 Table List of probes altered by social stress—time course analysis. (XLSX)

S4 Table Extended list presenting quantitative qPCR validation of microarray data. (XLSX)

S5 Table Functional annotation of genes altered by social stress. (XLSX)

S6 Table BioGPS tissue gene expression. (XLSX)

S7 Table ARRIVE guidelines checklist. (XLSX)
