# Supplementary figures and images for: The Effect of Acute and Chronic Social Stress on the Hippocampal Transcriptome in Mice
Source: PLoS One. 2015 Nov 10;10(11):e0142195. doi: 10.1371/journal.pone.0142195 (PMC4640871; doi:10.1371/journal.pone.0142195)

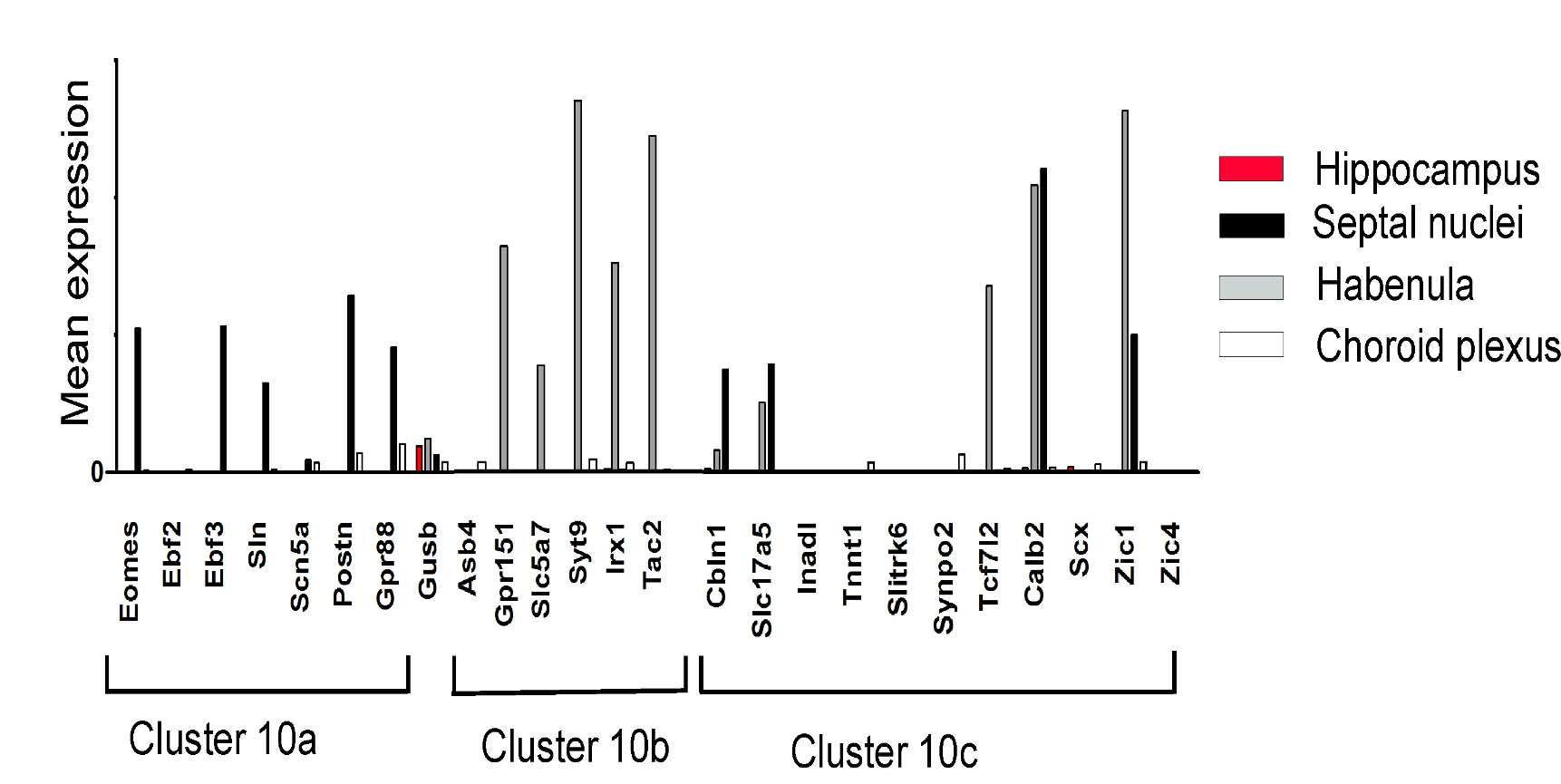

Supplement: S1 Fig — The intensity of gene expression in the Allen Brain Atlas is coded originally by colors. The average level of each transcript is expressed as a sum of mean intensities for blue, green and red channel multiplied by an integer representing different levels of expression in the Allen Brain Atlas. (TIF) [file pone.0142195.s001.tif]
